# Supplementary material for: Comparative multiomics analysis of cell physiological state after culture in a basket bioreactor
Source: Sci Rep. 2022 Nov 23;12:20161. doi: 10.1038/s41598-022-24687-4 (PMC9686226; doi:10.1038/s41598-022-24687-4)
Supplement: Supplementary file 1 — Supplementary Information 1. [file 41598_2022_24687_MOESM1_ESM.zip › raw data/Transcriptomic raw data/6.Enrichment/2.KEGG/TE1vsTC1/ALL/src/csab04550.html]

csab04550


- csab:103223483

- Down regulated genes

103223483(-3.35312742084333)
- csab:103224207

- Down regulated genes

103224207(-1.32692055344416)

- csab:103219053

- Up regulated genes

103219053(2.46925485759509)

- csab:103224900

- Down regulated genes

103224900(-1.42998371610984)

- csab:103224900

- Down regulated genes

103224900(-1.42998371610984)

- csab:103246479

- Down regulated genes

103246479(-1.5490263767958)

- csab:103223483

- Down regulated genes

103223483(-3.35312742084333)
- csab:103224207

- Down regulated genes

103224207(-1.32692055344416)

- csab:103237430

- Up regulated genes

103237430(1.35562492669487)

- csab:103237430

- Up regulated genes

103237430(1.35562492669487)

Close
